# Home-based versus center-based care in children with cerebral palsy: a cost-effectiveness analysis

**Published:** 2015

**Authors:** E Sharif Azar, M Ravanbakhsh, A Torabipour, E Amiri, MH Haghighyzade

**Affiliations:** * Musculoskeletal Rehabilitation Research Center, Ahvaz Jundishapur University of Medical Sciences, Ahvaz, Iran; ** Department of Health Services Management, School of Health, Ahvaz Jundishapor University of Medical Sciences, Ahvaz, Iran; *** Department of Health, School of Health, Ahvaz Jundishapor University of Medical Sciences, Ahvaz, Iran

**Keywords:** cerebral palsy, home care, cost-effectiveness, quality of life

## Abstract

The rehabilitation services for children with cerebral palsy are provided in two forms: home-based care and center-based care. The aim of this research was to evaluate the cost-effectiveness of the home-based accordance with the center-based care for kids with cerebral palsy.

In this cost-effectiveness research, 56 children under 12 years old were assigned randomly to two rehabilitation programs: (1) clinic-based rehabilitation services (CBRS); and (2) home-based rehabilitation services (HBRS). Data were collected by two questionnaires: a strong life quality survey of children with cerebral palsy (CP QOL-Child) and medical and non-medical costs’ checklists. Finally, the incremental expense-efficacy rate (ICER) was used to determine the further expenses of one unit of the quality of life gained by CBRS compared with HBRS. The mean costs per patients for the home-based care group were less than the ones for the clinic-based care unit (US$ 660.3 vs. US$ 933.8). The costs of the rehabilitation services and transportation were the main costs in the two patients’ groups. The quality of life for cases in the home-based care group was better than the one of the clinic-based care team. The results showed that the home-based care method was more cost-effective than the centre-based care approach in children with cerebral palsy. The incremental cost-effectiveness ratio was calculated at about US$ 2.6.

The conclusion was that home-based care centers were more cost-effective than the centre-based care centers for children with cerebral palsy. Therefore, it was suggested that the health policy makers pay more attention to developing home-based care strategy in physically challenged children.

## Introduction

Cerebral palsy is the maximum typical illness condition in children [**1**]. Cerebral palsy (CP) is a term that refers to a group of non-progressive neurological disorders that may lead to bodily, mental and cognitive disorders [**2**,**3**]. Cerebral palsy is not a disease with a clear etiology [**4**]. Early signs of CP usually appear in childhood, but there is no agreement at the age of early signs of cerebral palsy [**5**,**6**]. Children with CP will have a variety of connected injuries, involving cognitive and body illness, sound and obvious deficiencies, nutritional and feeding difficulties, and respiratory diseases [**7**,**8**].

Also, children with CP may have a limitation in the performance of daily activities, societal participation and quality of life [**9**]. Prevalence of CP is 1.5 to 3.6 per every 1000 live births in the US [**10**]. A research reported that the incidence of CP was 2.1-2.4 at each 1,000 births in six countries [**11**].

Recent research has found that CP prevalence was 2.06 per 1000 live births in Iran [**12**,**13**]. Johnson reported that cerebral palsy was more common among males in Europe [**14**]. Cerebral palsy can restrict social functions, participation, and self-esteem of children [**15**,**16**]. Physical and cognitive disabilities can lead to social, economic and environmental problems for the patients with cerebral palsy [**17**-**21**]. 

Children with cerebral palsy may require specialized medical, rehabilitation, and social services [**22**]. The rehabilitation services for children with cerebral paralysis were provided in two forms: home-based care and center-based care. The centre-based care may increase non-medical costs such as transportation costs [**23**]. Another type of services for CP patients is home-based care. Home-based care has advantages including improving the access to services, reducing the waiting time to receive services, increasing the patient’s safety, improving the active participation of the families to manage their patients [**13**,**24**-**26**]. In some cases, home-based care may be expensive for CP patients [**27**,**28**]. In Iran, rehabilitation services were mainly provided by private clinics. Iranian people pay more than 50% of the medical and paramedical charge as out of pocket costs [**26**]. Therefore, disabilities such as cerebral palsy can impose a high economic burden on the patients and their families. Also, families with physically challenged children need more psycho-social considerations [**29**]. 

A study showed that the average cost per CP patient was $ 43,431 in Australia [**30**]. Some studies reported results about costs of disability in children [**31**,**32**]. The high costs of these disabilities reveal the need for health insurance coverage for physically challenged patients [**33**]. Therefore, the primary concern of policy-makers and community is whether home-based care strategy is a cost-effective alternative for physically challenged patients. Currently, home-based care centers have been implemented in different countries. However, there are not many pieces of evidence whether this strategy is more cost effective (Bentur 2001) [**11**]. 

The cost-effectiveness study is now one of the most standardized tools of economic evaluation in the health sector [**13**,**34**]. Estimations of expenses, treatment impacts, and cost-effectiveness ratio supply obvious instruction to decision making by policy makers [**35**]. This research objected to compare cost-efficacy of home-based care and centre-based care methods in children with CP.

## Methods

**Study design and samples**

This is a cost effectiveness research. The study population consisted of children with CP. Data of 56 children with CP were collected randomly from May 2015 to July 2015 in Ahvaz, a metropolitan and southern city of Iran. Samples were assigned to two rehabilitation delivery programs: (1) clinic-based rehabilitation services (CBRS); and (2) home-based rehabilitation services (HBRS). In Iran, CP children refer to private rehabilitation centers. In this study, samples were selected from three rehabilitation centers. 

**Data gathering**

Data were collected by two questionnaires: a sound life quality survey of children with CP QOL-Child and non-medical and medical expenses checklists. The QOL-questionnaire included 66 items with seven domains. The responses assessed were based on a five-point Likert scale (from disagreeing to agree very much). The validity and reliability of CP QOL-child were confirmed by the similar study [**36**-**39**]. Data of non-medical and medical expenses including physiotherapy, occupational therapy, speech therapy, drug, individual visits, nursing care, diagnosis tests, radiology, injection, aid device, and transportation were gathered by a valid checklist [**40**].

In this study, indirect costs were not calculated for CP children. Studies showed that direct costs are the first costs of caring for children with CP (Control 2004) [**41**]. The average total cost per patient was calculated based on the sum of the non-medical and medical expenses. To reduce recall bias, data of the values were collected for previous three months by using an interview with parents. 

**Cost-effectiveness analysis**

The expense-efficacy and enhancement expense-efficacy rates were determined by the variation of expenses classified via the distinct of effectiveness (QOL-score). The enhancement expense-efficacy rate was determined by using the statement:

Expense A represents the costs of children with CP in centre-based care method; Cost B accounts for the expenses of children with CP at home-based care process. Effectiveness A accounts for the quality of life of children with CP in the centre-based care method, and Effectiveness B accounts for the quality of life of children with CP at home care. 

**Sensitivity Investigation**

Different scenarios were determined by main benefits changes. Data of inflation rate that were reported by the Iranian Central Bank (Inflation in Iran 2015) were the basis of variations of value parameter (-20 to +20% change in costs). The differences between values were determined by using a paired t-test. SPSS was used to analyze data.

## Results 

Out of 56 studied children, 75% of the children had spastic cerebral palsy. The results showed that more than half of the patients (51.7%) were male, and 76% were children under nine years old. Most patients were covered by the Iranian social security health insurance (ISSI) (**Table 1**).

**Table 1:** Descriptive characteristics of children with cerebral palsy

	Patients characteristics	Clinic	Home
		Percent (frequency)	Percent (frequency)
Age	4-6 years	14 (50)	4 (14.3)
	9-7 years	10 (35.7)	14 (50)
	10-12 years	4 (14.3)	10 (35.7)
Sex	male	15 (53.6)	14 (50)
	female	13 (46.4)	14 (50)
Type of CP	Spastic	21 (75)	21 (75)
	Ataxic	3 (10.7)	3 (10.7)
	Other	4 (14.3)	4 (14.3)
Medical insurance fund	Social Security fund	14 (50)	13 (46.4)
	Medical Services	7 (25)	6 (21.4)
	Iranian health	3 (10.7)	5 (17.9)
	Uninsured	4 (14.3)	4 (14.3)

The average cost of the uninsured patients was higher than that of patients who were insured by the health insurance funds in the two patients’ group. Also, the mean total cost per uninsured patient in the centre-based care group was greater than the one for another group (**Table 2**).

**Table 2:** The average cost of home and clinic-based care on insurance funds

Groups	Mean ± SD			
	Social Security insurance	The Medical Services insurance	Iranian Health insurance	Uninsured
Clinic (n = 28)	682.3 ±308.6	921.1 ±202.4	1000.1± 442.5	1775± 320.2
Home (n = 28)	662.7 ± 209.8	879 ± 125.7	907.5 ± 350.7	933.6 ± 396.5
Rial 29963 equal to US$ 1 (at the last officials exchange rate in November 2015)				

The findings demonstrated that there is a statistically clear distinction between the home-based care and the centre-based care group in terms of quality of life rate (P < 0.05). The average score of the quality of life for patients in the home-based care group was higher than the one for the patients in the centre-based care group, 404.1 ± 21.62 vs. 298.2 ± 50.89 respectively (**Table 3**).

**Table 3:** Domains of quality of life in homecare (n = 28) and clinic care (n = 28)

Domains of quality of life	Groups	Mean ± SD	Mean Difference	P-value
Social wellbeing and acceptance	Clinic	59.85 ± 11.74		
	Home	78.25 ± 6.96	-7.12	0.001
Functioning	Clinic	52.89 ± 12.93		
	Home	80.17 ± 6.77	-9.88	0.001
Participation and physical health	Clinic	54 ± 14.56		
	Home	73.5 ± 4.31	-6.79	0.001
Emotional wellbeing and self esteem	Clinic	29.1 ± 9.6		
	Home	38.75 ± 3.26	-5.03	0.001
Access to services	Clinic	45.64 ± 12.17		
	Home	70.42 ± 9.62	-8.44	0.001
Pain and impact of disability	Clinic	40.89 ± 10.35		
	Home	42.46 ± 6.33	0.68	0.49
Family Health	Clinic	15.89 ± 7.77		
	Home	20.57 ± 4.27	-2.79	0.001
Total mean score of quality of life	Clinic	298.2 ± 50.89		
	Home	404.1 ± 21.62	-10.12	0.001

According to **Table 4**, there is a statistically significant difference between home-based care and center-based treatment groups in terms of cost per patient (P < 0.001). The average cost per patient in center-based care and home-based treatment group was US$ 933.8 and US$ 660.3, respectively. The highest value assigned to occupational therapy services in both patients’ groups (US$ 7854.9 in home-based care vs. US$ 9060.7 in centre-based care). The results showed that the home-based care method was more cost-effective than the centre-based care process in children with cerebral palsy. According to the results of incremental cost-effectiveness ratio analysis (ICER), the cost per additional QOL in the centre-based care group was 2.58 times more than the one in the home-based-care group (**Table 5**). 

ICER=Cost A−Cost  BEffectiveness A−Effectiveness B=933.8−660.3298.2−404.1=2.58

Finally, the one-way sensitivity analysis showed that by reducing and increasing 20% of central cost units including Physiotherapy, Occupational Therapy, and Speech therapy services, no significant difference was found in the incremental cost-effectiveness ratio. Therefore, the results of CER were not sensitive to medical and rehabilitation costs (± 0.1%), but the results of CER were sensitive to transportation cost (± 11.9%) (**Table 6**).

**Table 4:** Difference in service costs between groups

	Service	clinic(N = 28)	Home (N = 28)	P-value
		Service cost (US$)	Service cost (US$)	
Medical	Physiotherapy	3180.6	3873.8	0.03
	Occupational Therapy	9060.7	7854.9	0.001
	Speech Therapy	2047.5	2509.1	0.007
	Drug	3111.5	882.7	0.28
	Aid Device	654.1	781	0.001
	Visits by specialist	1084.5	579.1	0.02
	Nursing Care	901.1	1081.2	1.21
	Diagnosis Tests	367.1	281.9	0.3
	Radiology	600.7	116.2	0.01
	Injection	40	17.9	0.001
Non-medical	Transportation	5098.2	512.2	0.008
	Total cost	26146	18490	0.001
	933.8	933.8	660.3	0.001
Rial 29963 equal to US$ 1 (at the last official exchange rate in November 2015)				

**Table 5:** The cost/ effectiveness ratios of the two groups

Groups	Cost	Effectiveness	C/ E	ICER
Clinic	933.8	298.2	3.1	-
Home	660.3	404.1	1.6	2.6

**Table 6:** A sensitivity analysis of cost-effectiveness

Variation	ICER		Change (%)
Initial scenario	2.6		
	Low value (-20%)	High value (20%)	
Rehabilitation services*	2.57	2.58	± 0.1%
Transportation	2.27	2.89	± 11.9%
* Physiotherapy, Occupational therapy, Speech therapy			

## Discussion 

The current research was objected to evaluate the cost-effectiveness of home-based accordance centre-based recovery equipment for children with cerebral paralysis. The results of the current research showed that the average direct cost of children with CP in the home-based care group was US$ 660.3; whereas the average cost of the services provided at the clinic was US$ 933.8 (P = 0.001) [**42**]. Lee and Koutkias et al. showed that the costs of home-based care are lower than the ones of centre-based care. Also, [**43**] Hoving and Novak found similar results. According to Wang et al., people with cerebral palsy related to other disabilities have a longer life expectancy, more care needs, more rehabilitation services, and costs. The findings of our research indicated that the cost of rehabilitation services, especially occupational therapy was the higher than the other services in children with CP. It should be noted that occupational therapy services are critical and fundamental services in children with CP. According to the findings of this research, all the medical and non-medical costs at the centre-based care group were more than the ones at the home-based care team. In addition, non-medical costs, including transportation in the centre-based care group, were about ten times higher than the ones in another group. According to Al-Oraibi et al., the cost of transportation is one of the initial costs for the parent of a child with CP. The findings showed that the costs of the uninsured patients were nearly twice the ones of the insured patients. The average costs of patients covered by the Social Security Insurance Fund (SSIF) were significantly less than the ones of other patients. The primary health insurance funds in Iran, including the Social Security fund, Iranian health Insurance (IHI), Armed Forces Medical Services Insurance (AFMSI) and Imdad (Relief) Committee Fund covers people, being compulsory. People covered by the necessary insurances pay 10 to 20% of deductible. The rehabilitation services in Iran are partially covered by primary health insurances. Therefore, out of pocket costs are increasing in some medical and rehabilitation services. This study showed that the mean total score of the quality of life is higher in home-based care teams than in other teams. The quality of life in children with CP involves moral, social, affectionate, and physical fitness.

A study showed that the home-based care services led to the reducing the restrictions in daily living actions with 28%. Studies showed that home-based care strategy leads to the improvement of the economic aspect, increasing the patients’ satisfaction, services quality, reducing medical errors, and traffic accidents. In some studies, the importance of family-centered-care approach is taken into consideration for the treatment of the child with cerebral palsy. Family-centered care can lead to an increase in the quality of life of children with CP and disability. The results showed that the home-based care method was more cost-effective than the centre-based care process in children with cerebral palsy. Studies indicated that home-based care is known as an effective care strategy and leads to the reducing of the cost and the increase in the quality of life.

## Conclusions 

We concluded that the home-based care was more cost-effective than the centre-based care in children with cerebral palsy. The results showed that the costs of rehabilitation services were higher than the other services in children with cerebral palsy. Therefore, it is suggested that the health policy makers should pay more attention to the development of home-based care strategy in physically challenged children by improving the required infrastructure such as subsidizing the costs of rehabilitation services.

**Acknowledgements**

The origin of information described in this article is from the Master project of Esmail Sharif Azar, Ahvaz Jundishapur University of Medical Sciences student; and financially supported by Ahvaz Jundishapur University of Medical Sciences (MSc Thesis grant no: PHT9412). The authors would like to thank the Research and Technology Deputy of Ahvaz Jundishapur University of Medical Sciences and the School of Rehabilitation Sciences for their financial and administrative support in undertaking the project. 

Ethical issues: IR.AJUMS.REC.1394. 277

Call the ethics committee that has approved the study.

Ahvaz Jundishapor University of Medical Sciences (AJUMS) committee

## References

[1] Imms C, Novak I, Kerr C (2015). Improving allied health professionals’ research implementation behaviors for children with cerebral palsy: protocol for a before-after study. Implementation Science.

[2] Rameckers E, Janssen-Patten Y, Essers I, Smeets R (2015). Efficacy of upper limb strengthening in children with Cerebral Palsy: A critical review. Research in Developmental Disabilities.

[3] Rosenbaum P, Paneth N, Leviton A, Goldstein M, Bax M, Damiano D (2007). A report: the definition and classification of cerebral palsy April 2006. Dev Med Child Neurol Suppl.

[4] Karen W, Krigger M (2006). Cerebral palsy: An overview. Am Fam Physician.

[5] Bosanquet M, Copeland L, Ware R, Boyd R (2013). A systematic review of tests to predict cerebral palsy in young children. Developmental Medicine & Child Neurology.

[6] McIntyre S, Morgan C, Walker K, Novak I (2011). Cerebral palsy—don't delay. Developmental Disabilities Research Reviews.

[7] Cooley WC (2004). Providing a primary care medical home for children and youth with cerebral palsy. Pediatrics.

[8] Novak I, Hines M, Goldsmith S, Barclay R (2012). Clinical prognostic messages from a systematic review on cerebral palsy. Pediatrics.

[9] Boyd (2013). Australian Cerebral Palsy Child Study: protocol of a prospective population-based study of motor and brain development of preschool aged children with cerebral palsy. BMC Neurology.

[10] Houtrow A, Kang  T, Newcomer R (2012). In-home supportive services for individuals with cerebral palsy in California. J Pediatr Rehabil Med.

[11] Soleimani F, Vameghi  R, Rassafiani M, Akbar Fahimi N (2011). Cerebral Palsy: Motor Types, Gross Motor Function and Associated Disorders. Iranian Rehabilitation Journal.

[12] Hirtz  D, Thurman D, Gwinn-Hardy  K, Mohamed  M, Chaudhuri  A, Zalutsky  R (2007). How common are the “common” neurologic disorders?. Neurology.

[13] Dalvand  H, Rassafiani  M, Hosseini  A, Khankeh  H, Samadi  A (2014 ). Challenge in handling children with cerebral palsy: A qualitative content analysis. Journal of Research in Rehabilitation Sciences.

[14] Johnson  A (2002 ). Prevalence and characteristics of children with cerebral palsy in Europe. Developmental Medicine & Child Neurology.

[15] Kirby RS, Wingate MS, Braun KVN, Doernberg NS, Arneson CL, Benedict RE (2011). Prevalence and functioning of children with cerebral palsy in four areas of the United States in 2006: a report from the Autism and Developmental Disabilities Monitoring Network. Research in Developmental Disabilities.

[16] Newman  CJ, O’Regan  M, Hensley  O (2006). Sleep disorders in children with cerebral palsy. Developmental Medicine & Child Neurology.

[17] Dalvand H, Rassafiani M, Hosseini SA, Samadi SA, Khankeh HR (2015). Concept Analysis of Occupational Therapy Handling in the Children with Cerebral Palsy: A Hybrid Model. Rehabilitation.

[18] Fedrizzi E, Pagliano E, Andreucci E (2003). Hand function in children with hemiplegic cerebral palsy: prospective follow-up and functional outcome in adolescence. Developmental Medicine & Child Neurology.

[19] Hemsley B, Balandin S, Worrall L (2012). Nursing the patient with complex communication needs: time as a barrier and a facilitator to successful communication in a hospital. Journal of Advanced Nursing.

[20] Missiuna C, Pollock N (1991). Play deprivation in children with physical disabilities: The role of the occupational therapist in preventing secondary disability. American Journal of Occupational Therapy.

[21] Specht J, King G, Brown E, Foris C (2002). The importance of leisure in the lives of persons with congenital physical disabilities. American Journal of Occupational Therapy.

[22] Wang B, Chen Y, Zhang J, Li J, Guo Y, Hailey D (2008). A preliminary study into the economic burden of cerebral palsy in China. Health Policy.

[23] Adler R, MacRitchie K, Engel GL (1971). Psychologic Processes and Ischemic Stroke (Occlusive Cerebrovascular Disease): I. Observations on 32 Men with 35 Strokes. Psychosomatic Medicine.

[24] Benton N (2001). Hospital at home: what is its place in the health system?. Health Policy.

[25] McBride  KL, White  CL, Serial  R, Mayo  N (2004). Postdischarge nursing interventions for stroke survivors and their families. Journal of Advanced Nursing.

[26] Abolhallaje M, Hassani  S, Bastani  P, Ramezanian  M, Kazemi  M (2013). Determinants of catastrophic health expenditure in Iran. Iranian Journal of Public Health.

[27] De Kinderen  RJ, Postulart  D, Aldenkamp  AP, Evers  SM, Lambrechts  DA, de Louw  AJ (2015). Cost-effectiveness of the ketogenic diet and vagus nerve stimulation for the treatment of children with intractable epilepsy. Epilepsy Research.

[28] Kontodimopoulos  N, Niakas D (2008). An estimate of lifelong costs and QALYs in renal replacement therapy based on patients’ life expectancy. Health Policy.

[29] Sadat  AK, Saleh Made  H, Hemmati  S, Darvish  M, Heydari  ST, Tabrizi  R (2015). The Causal Factors Associated with the Loving Care of the Mothers of Children with Multiple Disabilities. International Journal of Community Based Nursing and Midwifery.

[30] Economics  A (2008). The economic impact of cerebral palsy in Australia 2007. Report for Cerebral Palsy Australia.

[31] Davidoff  AJ (2004). Insurance for children with special health care needs patterns of coverage and burden for families to provide adequate protection. Pediatrics.

[32] Hutton J, Colver A, Mackie P (2000). Effect of the severity of disability on survival in north east England cerebral palsy cohort. Archives of Disease in Childhood.

[33] Hwang  W, Weller W, Ireys  H, Anderson G (2001). Out-of-pocket medical spending for care of chronic conditions. Health Aff.

[34] Eichler HG, Kong SX, Gerth WC, Mavros P, Jönsson B (2004). Use of Cost-Effectiveness Analysis in Health-Care Resource Allocation Decision-Making: How Are Cost-Effectiveness Thresholds Expected to Emerge?. The value in health.

[35] Marseille E, Larson B, Kazi DC, Kahn JG, Rosen S (2015). Thresholds for the cost–effectiveness of interventions: alternative approaches. Bull World Health Organ.

[36] Chen  K-L, Tseng M-H, Shieh J-Y, Lu L, Huang C-Y (2014). Determinants of quality of life in Children with Cerebral Palsy: A comprehensive biopsychosocial approach. Research in developmental disabilities.

[37] Sam  K-L, Wang H-Y, Li  C, Lo SK (2013). Item hierarchy of the Chinese version of cerebral palsy quality of life for children. European Journal of Pediatric Neurology.

[38] Soleimani  F, Vameghi  R, Kazemnejad  A, Fahimi  NA, Nobakht  Z, Rassafiani  M (2015). Psychometric Properties of the Persian Version of Cerebral Palsy Quality of Life Questionnaire for Children. Iranian Journal of Child Neurology.

[39] Waters  E, Davis E, Mackinnon A, Boyd  R, Graham HK, Kai Lo S (2007). Psychometric properties of the quality of life questionnaire for children with CP. Developmental Medicine & Child Neurology.

[40] Torabipour A, Asl ZA, Majdinasab N, Ghasemzadeh R, Tabesh H, Arab M (2014). A Study on the Direct and Indirect Costs of Multiple Sclerosis Based on Expanded Disability Status Scale Score in Khuzestan, Iran. International Journal of Preventive Medicine.

[41] (2004). Economic costs associated with mental retardation, cerebral palsy, hearing loss, and vision impairment--the United States, 2003. MMWR Morbidity and mortality weekly report. Control CfD, Prevention.

[42] Lee TW (2004). Economic evaluation of visiting nurse services for the low-income elderly with long-term care needs. Taehan Kanho Hakhoe Chi.

[43] Hoving M, Evers S, Ament A, Van Raak E, Valles J (2007). Intractable spastic cerebral palsy in children: A Dutch cost of illness study. Developmental Medicine & Child Neurology.

